# Rapid Computer‐Aided Diagnosis of Stroke by Serum Metabolic Fingerprint Based Multi‐Modal Recognition

**DOI:** 10.1002/advs.202002021

**Published:** 2020-09-23

**Authors:** Wei Xu, Jixian Lin, Ming Gao, Yuhan Chen, Jing Cao, Jun Pu, Lin Huang, Jing Zhao, Kun Qian

**Affiliations:** ^1^ State Key Laboratory for Oncogenes and Related Genes Division of Cardiology Renji Hospital School of Medicine Shanghai Jiao Tong University 160 Pujian Road Shanghai 200127 P. R. China; ^2^ State Key Laboratory for Oncogenes and Related Genes School of Biomedical Engineering Shanghai Jiao Tong University Shanghai 200030 P. R. China; ^3^ Department of Neurology Minhang Hospital Fudan University 170 Xinsong Road Shanghai 201199 P. R. China; ^4^ School of Management Science and Engineering Dongbei University of Finance and Economics Dalian 116025 P. R. China; ^5^ Center for Post‐doctoral Studies of Computer Science Northeastern University Shenyang 110819 P. R. China; ^6^ Stem Cell Research Center Renji Hospital School of Medicine Shanghai Jiao Tong University 160 Pujian Road Shanghai 200127 P. R. China

**Keywords:** deep learning, diagnostics, mass spectrometry, metabolic fingerprints, stroke

## Abstract

Stroke is a leading cause of mortality and disability worldwide, expected to result in 61 million disability‐adjusted life‐years in 2020. Rapid diagnostics is the core of stroke management for early prevention and medical treatment. Serum metabolic fingerprints (SMFs) reflect underlying disease progression, predictive of patient phenotypes. Deep learning (DL) encoding SMFs with clinical indexes outperforms single biomarkers, while posing challenges with poor prediction to interpret by feature selection. Herein, rapid computer‐aided diagnosis of stroke is performed using SMF based multi‐modal recognition by DL, to combine adaptive machine learning with a novel feature selection approach. SMFs are extracted by nano‐assisted laser desorption/ionization mass spectrometry (LDI MS), consuming 100 nL of serum in seconds. A multi‐modal recognition is constructed by integrating SMFs and clinical indexes with an enhanced area under curve (AUC) up to 0.845 for stroke screening, compared to single‐modal diagnosis by only SMFs or clinical indexes. The prediction of DL is addressed by selecting 20 key metabolite features with differential regulation through a saliency map approach, shedding light on the molecular mechanisms in stroke. The approach highlights the emerging role of DL in precision medicine and suggests an expanding utility for computational analysis of SMFs in stroke screening.

## Graphical abstract

Rapid extraction of serum metabolic fingerprints (SMFs) is achieved using nano‐assisted laser desorption/ionization mass spectrometry, consuming 100 nL of native serum in seconds. Further integrating SMFs with clinical indexes by deep learning, the SMF based multi‐modal recognition achieves noninvasive diagnosis of stroke. As a platform approach, it can be adapted to many medical modalities and disease applications in the near future.

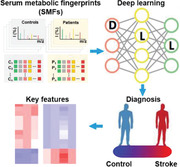

Stroke is a leading cause of disability and mortality worldwide.^[^ ^1^ ^]^ Over 50% of the stroke survivors suffer from a permanent handicap.^[^ ^2^ ^]^ Rapid diagnosis of stroke is critical to offer acute intervention in 3–6 h, which is the upper limit of the treatment window to achieve the best long‐term outcomes.^[^ ^3^ ^]^ While the current imaging‐based detection approaches of stroke can achieve rapid test, the diagnostic performance is limited in clinical use.^[^ ^4^ ^]^ For instance, computerized tomography may remain normal in patients with mild stroke, providing low sensitivity of ≈30%.^[^ ^5^ ^]^ In comparison, magnetic resonance imaging (MRI) is more sensitive (sensitivity of >80%), but not feasible for restless patients (≈20–79% of all stroke patients) with noncompliance and not accessible in remote and undeveloped areas (≈€0.5–2.5 million per device).^[^ ^6^ ^]^ Managing stroke requires a novel diagnostic method as an adjunct to the existing imaging modalities, with high speed, precise detection, and cost‐effective assays.

Molecular analysis shed light on the therapeutic decision making in patients with symptoms of stroke, including proteins and metabolites.^[^ ^7^ ^]^ The detection of protein biomarkers relies on enzyme‐linked immunosorbent assay, offering unsatisfactory sensitivity and specificity, long antibody–antigen reaction, and expensive reagent costs.^[^ ^8^ ^]^ Compared to proteins, metabolites enable a closer association with the disease phenotype, to characterize the complicated pathological and physiological process for diagnostic purpose.^[^ ^9^ ^]^ The current methods for metabolite detection rely on fluorescence, electrochemistry, and mass spectrometry (MS). Notably, the fluorescent labels are required to tag certain metabolites in fluorescence detection which will distort the normal function of metabolites,^[^ ^10^ ^]^ while electrochemistry is indirect by studying the relationship between oxidation/reduction chemistry and targeted metabolites.^[^ ^11^ ^]^ In addition, both approaches above fail to detect various metabolites in a single experiment. Compared to the fluorescence and electrochemistry methods, MS shows the pros as the parallel extraction and measurement of molecular information, becoming the primary method to detect untargeted metabolites.^[^ ^12^ ^]^ However, the bioapplication of MS is hindered with the cons of tedious pretreatment, concerning the high sample complexity and low metabolite abundance in real‐case biosamples.^[^ ^9^, ^13^ ^]^ With the development of nanotechnology,^[^ ^14^ ^]^ the recently developed nano‐assisted laser desorption/ionization (LDI) MS serves as a practical tool for metabolic analysis, due to the high analysis throughput (≈300 samples h^−1^) and accurate metabolic identification (mass error <50 ppm).^[^ ^15^ ^]^ In contrast to proteins, the serum metabolic fingerprints (SMFs) extracted by nano‐assisted LDI MS may become better alternatives towards rapid and cost‐effective diagnostics for stroke in an antibody‐free manner.

A systematical model integrating SMFs with clinical accessible data (e.g., clinical indexes) outperforms a single biomarker or a panel of selected biomarkers.^[^ ^16^ ^]^ The occurrence and development of diseases involve complex regulation mechanisms, hampering the analysis of pathogenic factors by using single‐modal data.^[^ ^17^ ^]^ The path of technological progress now enters the era of multiple modalities, which could be used to depict biosystems' strategies under chemical or environmental stress with an immediate, dynamic, and comprehensive picture.^[^ ^18^ ^]^ Conventional routes failed to couple SMFs and other data for clinical use, due to the inconsistent dimensions of multiple modalities and intrinsic heterogeneity of biological system.^[^ ^19^ ^]^ Therefore, it is of key significance to construct an integrative SMF based multi‐modal recognition for the sake of precise diagnostic use.

Deep learning (DL) can scale to large data sets and accept multiple data types as input, thus holding promise in heterogeneous healthcare data.^[^ ^20^ ^]^ DL as a subfield of machine learning (ML), has emerged as an important analysis tool with enhanced computational power and shattered performance benchmarks in various applications.^[^ ^21^ ^]^ In particular, the healthcare and medicine stand to benefit from DL, but still pose challenges with poor prediction mechanisms to interpret by selecting significant features through classification.^[^ ^22^ ^]^ Common feature selection in DL relies on salient region maps to guide attention and gaze to the most conspicuous regions in a visual scene, by detecting salient regions in the images based on feature integration theory. The concept of saliency map was first proposed to represent the conspicuity of a location in a visual scene and then achieved in a manner of a computational model to solve complex scene understanding problems in various fields, including neuroscience, psychology, and medical diagnosis, etc.^[^ ^23^ ^]^ However, the saliency‐driven approach has not been verified in mass spectrometric data concerning the large multi‐dimensions of SMFs, while is only specific to image classification with figures as inputs.^[^ ^23^, ^24^ ^]^ In this regard, an advanced DL model remains to be explored, to both achieve integrative modal fusion of SMFs with other clinical data and enable feature selection with interpretable saliency.

Using recent advances in nano‐assisted LDI MS for metabolic fingerprinting, we developed a new platform that overcame the major challenges in rapid, clinically acceptably accurate, and low‐cost diagnosis of stroke. We analyzed 100 nL of native serum (**Figure** **1**a) in seconds (Figure 1b), to directly extract SMFs (Figure 1c) from stroke patients and healthy controls by nano‐assisted LDI MS. As a result, we performed DL of SMFs to identify stroke with area under curve (AUC) of 0.738 for discovery cohort and 0.803 for validation cohort (Figure 1d). Through the combination of SMFs with clinical indexes by DL for multi‐modal recognition, we screened stroke with AUC of 0.845 for validation cohort (Figure 1e). In addition, we selected a panel of 20 key metabolite features from classification and may serve as potential biomarkers for stroke diagnosis. We proposed a rapid and low‐cost approach as an adjunct to the existing imaging modalities and aided in better management for stroke in clinics.

**Figure 1:**
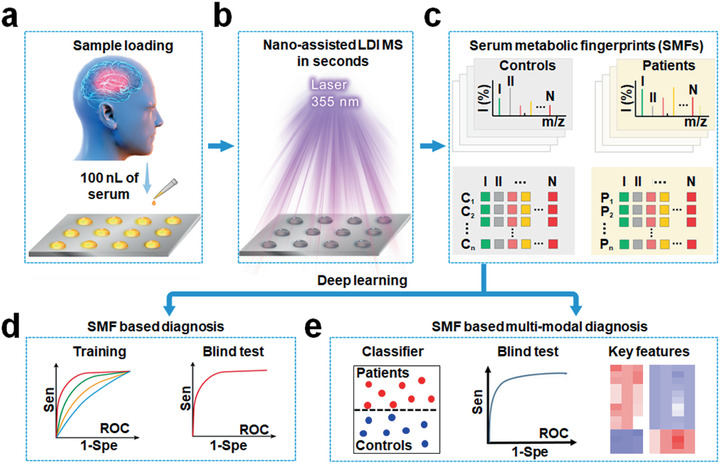
Overall schematics for extraction of serum metabolic fingerprints (SMFs) towards stroke diagnosis by deep learning (DL). a) Only 100 nL of native serum was directly loaded in a microarray without any labeling, derivatization, and chromatography procedures. Then, b) a Nd:YAG laser (355 nm) was irradiated onto the analyte microarray, facilitating nano‐assisted desorption/ionization (LDI) process to obtain raw mass spectra. c) SMFs were extracted from the discovery cohort and validation cohort for downstream analysis. DL algorithm was employed to achieve direct d) SMF based diagnosis by using only SMFs as inputs and e) SMF based multi‐modal diagnosis by integrating SMFs with clinical indexes. The classification performances were evaluated from discovery cohort and validation cohort. Key metabolite features were selected by DL based saliency map approach.

We constructed nano‐assisted LDI MS (Figure 1a) to record SMFs from serum, towards the downstream SMF based single‐ (Figure 1d) and multi‐modal diagnosis (Figure 1e).^[^ ^15^, ^16^ ^]^ We randomly split all serum samples into nonoverlapping discovery and independent validation (≈4:1) cohorts, to avoid overfitting and optimistic estimates of generalization accuracy. Specifically, the demographic data of healthy controls and stroke patients for the discovery (*n* = 275, 137/138) and blind (*n* = 69, 35/34) cohorts were presented in **Figure** **2**a. No significant differences of age and sex were observed between two groups in the discovery cohort (*p* > 0.05, by Student's t‐test and Chi‐square test; Table S1, Supporting Information). Prior to the large cohort analysis, we performed power analysis to estimate the effect sample size with statistical power as 0.9 at a significance level of 0.15, demonstrating a minimum number of 62 samples is enough to conclude the statistical meaningful results.^[^ ^25^ ^]^ Clinical indexes (see Table S2 in the Supporting Information for detailed baseline characteristics) were required from clinics, including *γ*‐glutamyl transferase, albumin, and albumin/globulin ratio, etc. Sample groups were comparable for most clinical indexes (e.g., albumin, *p* > 0.05, by Student's t‐test).

**Figure 2:**
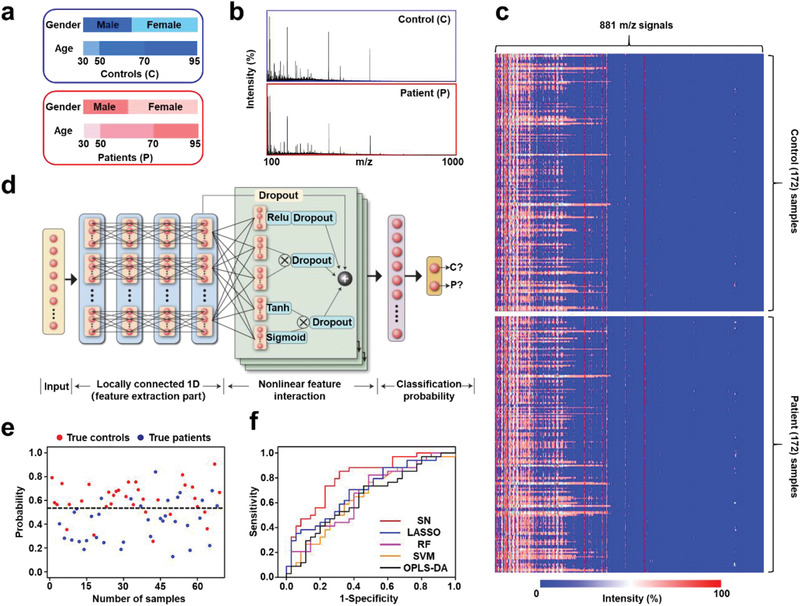
Diagnosis of stroke by SMF based diagnosis with DL. a) Demographics of 344 clinical samples as the discovery cohort (blue) and validation cohort (red). The age and gender of different groups were matched with no significant difference (*p* > 0.05). b) Typical mass spectra from a healthy control and a stroke patient by nano‐assisted LDI mass spectrometry (MS), with *m*/*z* ranging from 100 to 1000 Da. c) SMFs were extracted from raw mass spectra of 172 healthy controls and 172 stroke patients, each containing 881 *m*/*z* signals. d) Stroke network (SN) layout. The SN consisted of i) input data using SMFs (yellow), ii) four locally connected 1D layers as feature extraction part (blue), iii) stacked nonlinear feature interaction layer providing additional nonlinear transformations (green), and iv) softmax classification layer that models SMFs‐to‐disease data for classification probability output (pink). e) A sample‐level plot stratifying healthy controls (red) and stroke patients (blue) for stroke screening in validation cohort (*n* = 69; 35/34, control/patients). The black dashed line indicated the threshold, determined by maximized the Youden index in the discovery cohort. f) Receiver operating characteristic (ROC) curves using SN (red), least absolute shrinkage and selector operator (LASSO, blue), random forest (RF, pink), support vector machine (SVM, orange), and orthogonal partial least squares discriminant analysis (OPLS‐DA, black) to distinguish between controls and stroke patients in validation cohort (*n* = 69; 35/34, control/patients).

Using nano‐assisted LDI MS, we recorded the typical mass spectra consuming 100 nL of serum from a healthy control and a stroke patient (Figure 2b). To investigate the reproducibility at different sample volumes, we extracted and compared SMFs from 50/100/150/200/500/1000 nL of native serum, by the nano‐assisted LDI MS (Figure S1, Supporting Information). A frequently used cosine correlation method was employed to investigate the spectra similarities among different SMFs, showing high reproducibility with spectra similarity of 0.987–0.996 over the range of 50–200 nL.^[^ ^26^ ^]^ Further increasing the sample consumption to 500 and 1000 nL, the spectra similarity decreased to 0.850 and 0.808 respectively, due to the influenced laser absorption by thick deposition of serum sample.^[^ ^27^ ^]^ We extracted 881 *m*/*z* signals (as SMFs) from raw mass spectra and built the blueprint showing signal uniformity for each sample (Figure 2c). The above results demonstrated reliability and application potency of direct serum metabolic fingerprinting for diagnostics. Notably, tedious sample pretreatment (e.g., derivatization) and lengthy chromatographic separation (≈40 min) are required to reduce biosample complexity and enrich target molecules in conventional MS, including liquid chromatography‐mass spectrometry and gas chromatography‐mass spectrometry.^[^ ^28^ ^]^ In contrast to the conventional MS, our approach avoided the sample treatment procedure, suitable for large scale clinical use with enhanced analytical speed (approximately seconds), reduced sample consumption (down to ≈nL), and low cost (≈€3 per test).^[^ ^15^, ^29^ ^]^

To test the classification performance of SMFs, we utilized deep neural networks algorithm (Figure 2d) which was suitable for big data‐based classification tasks, to develop a DL model (denoted as stroke network (SN)) for the diagnosis of stroke.^[^ ^30^ ^]^ We validated the effectiveness of the model using ten‐fold cross‐validation. The average performance of the model was evaluated by sensitivity and specificity, as well as AUC. As a result, for the discovery cohort, we achieved mean sensitivity of 71.01% and specificity of 75.18% with AUC of 0.738 (Figure S2, Supporting Information). For the independent validation cohort, we achieved sensitivity of 73.53% and specificity of 77.14% with AUC of 0.803 (Figure 2e,f). Notably, we adjusted the number of discovery subjects from 70 to 275 and obtained an increasing AUC with enhanced performance (Figure S3, Supporting Information). The improved diagnostic performance can be attributed to the increased sample information as input to train a precise classifier, consistent with previous reports.^[^ ^31^ ^]^ In addition, we investigated the difference by optimizing the iteration numbers (from 1 to 100, Figure S4, Supporting Information). After 80 epochs (iterations through the entire dataset), the training was stopped due to the absence of further improvement in accuracy and the gradual increase of cross‐entropy loss. We further performed permutation test and concluded there were no overfitting when using SN, with *p* = 0.001 from permutation test (Figure S5, Supporting Information)^[^ ^32^ ^]^ and consistency between validation (AUC = 0.803) and discovery set (AUC = 0.738).^[^ ^33^ ^]^ The non‐overfitting modeling can be attributed to the dropout step, the early‐stop approach, and the nonoverlapping sample splitting in SN. For the dropout step, we introduced a unique dropout procedure (Figure 2d) into the SN, to randomly deactivate certain units (along with their connections) during training. As a result, the partly deactivated SN significantly avoided overfitting by decreasing the algorithm redundancy.^[^ ^34^ ^]^ For early‐stop, the training in SN stopped early after 80 epochs, preventing the potential overfitting from overoptimization of diagnostic performance.^[^ ^35^ ^]^ For the nonoverlapping sample splitting, the whole samples were split into nonoverlapping discovery and validation set, which is universally employed to avoid information leakage during each training step and prevent overfitting.^[^ ^33^, ^36^ ^]^ By contrast, we assessed the diagnostic accuracy of the major clinical used ML algorithms under the same protocol, including least absolute shrinkage and selector operator (LASSO; AUC: 0.703 for validation cohort, Figure 2f; Figure S3, Supporting Information and **Table** **1**), random forest (RF; AUC: 0.657 for validation cohort, Figure 2f; Figure S3, Supporting Information and Table 1), support vector machine (SVM; AUC: 0.653 for validation cohort, Figure 2f; Figure S3, Supporting Information and Table 1), and orthogonal partial least squares discriminant analysis (OPLS‐DA; AUC: 0.638 for validation cohort, Figure 2f, Figure S3, Supporting Information and Table 1). Notably, SN is superior with significantly higher performance for SMF based stroke diagnosis (*p* < 0.05, DeLong test).

**Table 1:** Comparison of diagnostic performance using different data inputs and algorithms

Input data	Algorithm^a)^	Cohorts	AUC [95% CI]^b)^	Sensitivity^c)^	Specificity^d)^
Clinical indexes	SN	Discovery	0.576 (0.508–0.643)	34.78%	70.80%
		Validation	0.703 (0.576–0.829)	67.65%	74.28%
Serum metabolic fingerprints (SMFs)	SN	Discovery	0.738 (0.678–0.799)	71.01%	75.18%
		Validation	0.803 (0.698–0.907)	73.53%	77.14%
	LASSO	Discovery	0.600 (0.533–0.667)	68.12%	53.29%
		Validation	0.703 (0.580–0.825)	70.59%	62.86%
	RF	Discovery	0.559 (0.491–0.626)	73.19%	32.12%
		Validation	0.657 (0.528–0.787)	82.35%	51.43%
	SVM	Discovery	0.502 (0433–0.571)	33.58%	66.42%
		Validation	0.653 (0.523–0.783)	76.47%	51.43%
	OPLS‐DA	Discovery	0.712 (0.651–0.773)	83.94%	44.20%
		Validation	0.624 (0.492–0.757)	74.29%	52.94%
SMF based multi‐modal data	CSN	Discovery	0.739 (0.679–0.800)	74.64%	72.26%
		Validation	0.845 (0.745–0.944)	88.24%	80.00%
Metabolite features	SN	Discovery	0.711 (0.648–0.773)	75.36%	69.34%
		Validation	0.790 (0.681–0.899)	73.53%	80.00%

a) SN: stroke network, LASSO: least absolute shrinkage and selector operator, RF: random forest, SVM: support vector machine, OPLS‐DA: orthogonal partial least squares discriminant analysis, CSN: clinical stroke network;

b) AUC referred to area‐under‐the‐curve from receiver operation curves (ROC);

c) Sensitivity referred to the ratio of the number of true positives to the total number of patients;

d) Specificity referred to the ratio of the number of true negatives to the total number of controls.

The higher performance of SN can be attributed to the end‐to‐end learning and multi‐layer stacked network, compared to conventional ML algorithms that have been used universally. For end‐to‐end learning, the feature extraction and disease classification in SN were performed in one step, computing convolutional and nonlinear transforms of the SMFs to retrieve high‐level abstractions for the final classification stage of the network. In comparison, separate feature extraction and supervised classification procedure are required to be integrated into a reliable routine step‐by‐step in the conventional ML algorithms, which is challenging when high‐dimensional SMFs involved (≈881 *m*/*z* signals). For multi‐layer stacked network, the locally connected 1D layer (Figure 2d, blue) and stacked nonlinear feature interaction layer (Figure 2d, green) were constructed in SN to address the linearity and nonlinearity among input features (≈881 *m*/*z* signals). In particular, the nonlinear feature interaction layer avoided potential overfitting and improved classification performance under limited samples, compared to multi‐layer perception (a universally used architecture of neural network). In comparison, conventional ML algorithms fail to address the nonlinear interaction among features, either with ignoration in LASSO, SVM, and OPLS‐DA or requiring a high‐order interactive RF.^[^ ^37^ ^]^

Clinical informative markers should further improve diagnostic performance. We integrated both SMFs and clinical indexes (Figures S6 and S7, Supporting Information) into a single unified prediction framework. We developed a clinical SN (CSN), by incorporating clinical indexes into the fully connected layers of the SN (**Figure** **3**a). Both data were presented to the network during training, enabling clinical indexes to influence the patterns learned by CSN to provide global and systematical bioinformation. We repeated our experiments using CSN trained on equivalent sample sets. The SMF guided multi‐modal recognition achieved mean AUC of 0.739 for discovery cohort (sensitivity: 74.64%, specificity: 72.26%; Figure 3b) and 0.845 for validation cohort (sensitivity: 88.24%, specificity: 80.00%; Figure 3c,d, Table 1). In comparison, we built a single‐modal model on clinical indexes only, yielding mean AUC of 0.576 (Figure 3e, Table 1) with sensitivity of 34.78% and specificity of 70.80% for discovery cohort; and mean AUC of 0.703 with sensitivity of 67.65% and specificity of 74.28% for validation cohort (Figure 3f,g, Table 1). In particular, any individual clinical index failed to offer the precise diagnosis for clinical use with AUC ranging from 0.328 to 0.632 (Table S3, Supporting Information), including those have been reported as potential serum biomarkers for stroke (e.g., creatine kinase isoenzymes, homocysteine, uric acid, creatinine, and hemoglobin).

**Figure 3:**
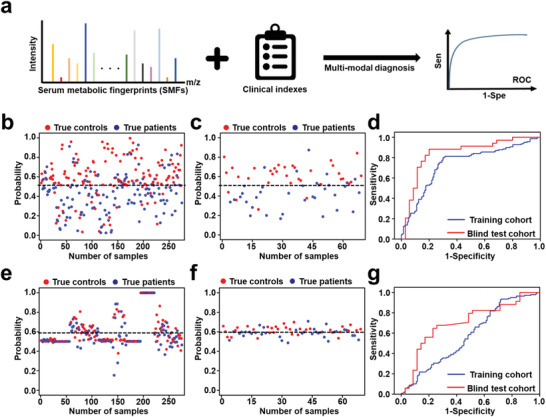
Diagnosis of stroke by SMF based multi‐modal diagnosis with DL. a) Schematic workflow for the integration of SMFs with clinical indexes for multi‐modal recognition (clinical stroke network (CSN)). Diagnosis by SMF based multi‐modal data (CSN) as input, including a sample‐level plot stratifying healthy controls (red) and stroke patients (blue) for b) discovery cohort (*n* = 275; 137/138, controls/patients) and c) validation cohort (*n* = 69; 35/34, controls/ patients), and d) ROC curves showing the diagnostic performance of input data in discovery (blue) and validation (red) cohort. Diagnosis by clinical indexes as input, including a sample‐level plot stratifying healthy controls (red) and stroke patients (blue) for e) discovery cohort (*n* = 275; 137/138, controls/patients) and f) blind test cohort (*n* = 69; 35/34, controls/patients), and g) ROC curves showing the diagnostic performance of input data in discovery (blue) and validation (red) cohort. The black dashed line in (b,c) and (e,f) indicated the threshold, determined by maximized the Youden index in the discovery cohort.

Notably, the multi‐modal recognition by CSN provided enhanced diagnostic performance, compared to the single modal recognition based on clinical indexes only by SN (*p* < 0.05, DeLong test). Further compared to brain imaging methods (e.g., MRI, the gold standard in clinics) that required expensive equipment, the SMFs guided multi‐modal approach by CSN featured higher patient compliance (only consuming 100 nL of serum) and cost‐effective detection (≈€3 per test) to be a valuable adjunct for imaging methods towards stroke screening in clinics, especially those from remote or undeveloped areas.

To select the key important metabolite features, we conducted feature selection on 881 *m*/*z* signals. We constructed an advanced saliency map approach to achieve the DL‐based variable selection (see details in Supporting Information). We identified 20 *m*/*z* signals derived from the discovery datasets as the key metabolite feature panel (Table S4, Supporting Information). We observed upregulation for *m*/*z* 149.07 and other 9 *m*/*z* signals (Figure S8, Supporting Information). In parallel, we observed downregulation for *m*/*z* 159.07 and other 9 *m*/*z* signals (Figure S8, Supporting Information). We further studied the altered pathways related to the above metabolite feature panel by pathway analysis in MetaboAnalyst (http://www.metaboanalyst.ca/), suggesting six differential permutated pathways in stroke patients (Figure S9 and Table S5, Supporting Information). The pathway analysis based on key metabolites is critical for interpreting the underlying pathological mechanisms of stroke. Notably, clear classifications in discovery (AUC of 0.711, Figure S10a,b, Supporting Information) and validation (AUC of 0.790, Figure S10c,d, Supporting Information) cohorts were observed using the 20 *m*/*z* signals as inputs, indicating that the metabolite feature panel could distinguish stroke patients from healthy controls. The results demonstrated that the 20 *m*/*z* signals performed well and may facilitate the simple analysis and large‐scale application of our approach towards real diagnostics.

It is well known that the poor interpretability of “black‐box” DL models remains a significant barrier for the validation and adoption of DL in clinics. The conventional feature selection in DL (e.g., feature extraction and transformation) fails in SMF based multi‐modal system (a typical “small n large p” case), concerning 1) the multi‐collinearity among 881 *m*/*z* signals (as input) and 2) high dimensionality (881 *m*/*z* signals) compared to small subjects (344 samples). Saliency map approach has been developed to address the proposed challenges and detect discriminant regions, while is currently specific to graphics as inputs. Specifically, SMFs can be viewed as structured data points on a predefined grid by *m*/*z* bins, similar to images on grid by pixels. Inspired by saliency computation in image classifications, we converted SMFs into two‐dimensional graphics (Figure S11a,b, Supporting Information) and for the first time computed the saliency map (Figure S11c, Supporting Information) for SMF based multi‐modal system, facilitating the efficient feature selection. Notably, discriminating the very similar spectra (Figure 2b) within SMFs is challenging using conventional feature selection approaches. In comparison, our modified saliency map approach showed significant differences which could be intuitively distinguished with different color shown in the colormaps (Figure S11a,b, Supporting Information). The designed saliency map approach benefitted the feature selection of large hyperspectral data, due to the simplified biomarker identification and quantifiable visualization results. For the simplified biomarker identification, the SMFs for each sample were transformed into a two‐dimensional image, providing a more obvious display of the relative intensity of *m*/*z* features in the colormap over the original spectra and thus simplifying the identification of potential biomarkers. For the quantifiable visualization results, the average value of saliency map at each point was utilized for quantifying, categorizing, and representing the significance of input features within SMFs. Feature selection provides insight into the biological patterns associated with increased risk and may serve as a practical tool to guide further exploration of molecular mechanisms in stroke.

As a limitation, further investigation is required to enhance the diagnostic accuracy by increasing the sample size and to verify the clinical adaptability by introducing prospective cohorts of patients presenting with stroke and stroke‐mimic symptoms. In addition, the proposed unified diagnostic framework is not exclusively specific to stroke applications. Therefore, more attempts should be adapted to other medical modalities and disease applications.

In summary, we constructed a binary classification model (SN) based on SMFs and clinical indexes by DL, which provided enhanced diagnostic outcomes compared to the reported single serum biomarker paradigm for differentiating stroke against healthy controls with fast analysis and low cost. Regarding a lack of efficient biomarkers in stroke thus far, the availability of the multi‐modal recognition might facilitate the “real‐time” rapid diagnosis (in seconds) and clinical management of stroke. The present multi‐modal recognition promoted a new liquid biopsy tool for rapidly diagnosing stroke, in a label‐free manner. We anticipated the design of computer‐aided metabolic protocols could easily translate into the clinical workflow in the near future.

## Experimental Section

All experimental information is provided in the Supporting Information, including clinical subject characteristics, serum harvesting, nano‐assisted LDI MS, deep learning method, and machine learning method, etc. All the investigation protocols in this study were approved by the institutional ethics committees of the School of Biomedical Engineering, Shanghai Jiao Tong University (SJTU) and Central Hospital of Minhang District, Shanghai. All subjects provided written informed consent to participate in the study and approved the use of their serum samples for analysis, according to the ethical guideline of the 1975 Declaration of Helsinki.

## Conflict of Interest

The authors declare competing financial interest. The authors have filed patents for both the technology and the use of the technology to analyze bio‐samples.

## Supporting information

Supporting Information
