# Supplementary material for: Rapid Computer‐Aided Diagnosis of Stroke by Serum Metabolic Fingerprint Based Multi‐Modal Recognition
Source: Adv Sci (Weinh). 2020 Sep 23;7(21):2002021. doi: 10.1002/advs.202002021 (PMC7610260; doi:10.1002/advs.202002021)
Supplement: Supplementary file 1 — Supporting Information [file ADVS-7-2002021-s001.pdf]

© 2020 Wiley-VCH GmbH

ADVANCED SCIENCE
Open Access

Supporting Information

for *Adv. Sci.,* DOI: 10.1002/advs.202002021

Rapid computer-aided diagnosis of stroke by serum metabolic fingerprint based multi-modal recognition

Wei Xu, Jixian Lin, Ming Gao, Yuhan Chen, Jing Cao, Jun Pu, Lin Huang, Jing Zhao*, and Kun Qian **

© 2020 Wiley-VCH GmbH

Supporting Information

Rapid computer-aided diagnosis of stroke by serum metabolic fingerprint based multi-modal recognition

Wei Xu, Jixian Lin, Ming Gao, Yuhan Chen, Jing Cao, Jun Pu, Lin Huang, Jing Zhao*, and Kun Qian**

W. X., J. C., Prof. J. P., Prof. K. Q.
State Key Laboratory for Oncogenes and Related Genes, Division of Cardiology, Renji Hospital, School of Medicine, Shanghai Jiao Tong University, 160 Pujian Road, Shanghai, 200127, P. R. China
School of Biomedical Engineering, and Med-X Research Institute, Shanghai Jiao Tong University, Shanghai, 200030, P. R. China
E-mail: k.qian@sjtu.edu.cn

Prof. M. G., Y. C.
School of Management Science and Engineering, Dongbei University of Finance and Economics, Dalian, 116025, P. R. China
Center for Post-doctoral Studies of Computer Science, Northeastern University, Shenyang, 110819, P. R. China

Dr. J. L., Prof. J. Z.
Department of Neurology, Minhang Hospital, Fudan University, 170 Xinsong Road, Shanghai, 201199, P. R. China
E-mail: zhao_jing@fudan.edu.cn

Dr. L. H.
Stem Cell Research Center, Renji Hospital, School of Medicine, Shanghai Jiao Tong University, 160 Pujian Road, Shanghai, 200127, P. R. China
E-mail: linhuang@shsmu.edu.cn

Experimental Section

Clinical subject characteristics: A total of 344 subjects were consecutively recruited from January 2018 to August 2018 in Central Hospital of Minhang District, Shanghai, including 172 patients suffering stroke and 172 healthy controls undergoing routine health care maintenance. All patients were diagnosed by physical examination, blood tests, and urgent neuroimaging (e.g. computed tomography (CT) or magnetic resonance imaging (MRI)), according to the American Heart Association/American Stroke Association (AHA/ASA) guidelines.[1] Patients were excluded from the study if they had a history of other acute diseases or malignant diseases (e.g. cancer). Healthy controls with systematic diseases were also excluded from the study. Clinical indexes were acquired from hospital following the standardized protocols in clinics, including albumin, albumin/globulin ratio, white blood cell count (WBC), and alanine aminotransferase (ALT), etc.

We randomly assigned 344 subjects to (1) a discovery cohort of 138 stroke patients and 137 healthy controls; and (2) an independent validation cohort of 34 stroke patients and 35 healthy controls. The age and gender of stroke patients and healthy controls were matched with no significant difference for discovery cohort, for diagnostic purpose ($p > 0.05$, Table S1).

Serum harvesting: Whole blood samples were collected after overnight fasting (more than 8 hours), to eliminate the disturbance of diet. 3 mL of blood was drawn to BD Vacutainer SST tubes (Becton, Dickinson and Co., USA) and clotted at room temperature within 40 minutes. Serum samples were obtained by centrifuging at 5,000 rpm and 4°C for 10 minutes. After centrifugation, the precipitate was discarded and the supernatant serum was stored at -80℃

immediately (within 15 minutes).[2] The elapsed time was within 1 hour between blood draw, centrifugation, and ultimate storage at -80℃.

All the investigation protocols in this study were approved by the institutional ethics committees of the School of Biomedical Engineering, Shanghai Jiao Tong University (SJTU) and Central Hospital of Minhang District, Shanghai. All subjects provided written informed consent to participate in the study and approved the use of their serum samples for analysis, according to the ethical guideline of the 1975 Declaration of Helsinki.

Nano-assisted laser desorption/ionization mass spectrometry (LDI MS): For a typical nano-assisted LDI MS experiment, 100 nL of serum sample was pipetted on the stainless target plate. Then, 100 nL of matrix slurry was dropped over the analyte and dried as a thin layer for LDI MS analysis. Plasmonic nanoparticles were prepared and dispersed in water as the matrix in LDI MS, following the previous reports.[3] Mass spectra were collected as serum metabolic fingerprints (SMFs) in positive reflection mode using a Bruker autoflex speed TOF/TOF mass spectrometer (Bruker Daltonics, Bremen, Germany), equipped with a smartbeam™-II Nd:YAG laser at 355 nm (Figure 1) for high throughput applications (1,000 Hz repetition rates throughout all experiments). All mass spectra were collected at m/z range from 100 to 1,000 Da without any smoothing procedures. Five independent experiments for one sample were conducted to eliminate intra-individual deviation and improve the reproducibility and stability of the diagnosis results.

Deep learning (DL) method: The original input of both stroke network (SN) and clinical stroke network (CSN) was unified with 1024 dimensional input (x_input), in which 881 features for single-modal SMFs recognition including 881 m/z signals (x_spectral), and 905 features for SMFs based multi-modal recognition including 881 m/z signals (x_spectral) and 24 clinical indexes (x_ext). The rest features (143 for SMF based single-modal recognition and 119 for SMF based multi-modal recognition) were set as 0, to enhance the adaptability of the networks. All input features were centralized and scaled to (-1,1). The networks were designed based on the deep neural networks (DNN), with two main networks (feature extraction part (feature_extract) and non-linear feature interaction layer (feature_interaction). The reorganized features (96 both for SN and CSN) after extraction and interaction were input to the classification layer (Softmax) for classification probability output.

The detailed principle formula from the original 1024 dimensional input (x_input) to the softmax layer was as follows:

$$x\_input = \mathrm{concatenate}(x\_spectral, x\_ext) \quad (1)$$

$$x\_fs = \mathrm{feature\_extract}(x\_input) \quad (2)$$

$$x\_nl = \mathrm{feature\_interaction}(x\_fs) \quad (3)$$

$$y\_pred = \mathrm{softmax}(x\_nl) \quad (4)$$

The feature extraction part (feature_extract) was consisted of four locally connected 1D layers.[4] Each layer was subdivided into 32 regions for feature extraction. The four-layer feature extraction part was defined as:

$$X_l = \{X_l^{c1}, \ldots, X_l^{c32}\} \quad (5)$$

$$X_{l+1} = \{\mathrm{relu}(W^{c1} X_l^{c1}), \ldots, \mathrm{relu}(W^{c32} X_l^{c32})\} \quad (6)$$

where $X_l^{c1}, \ldots, X_l^{c32}$ represented 32 extracted regions in the l (l = 0, 1, 2, 3) layer, and $W^{c1}, \ldots, W^{c32}$ represented parameters based on Adam optimization algorithm in each 32 regions of discovery subject.

The non-linear feature interaction layer (feature_interaction) was used to analyze the non-linear relationship among input features. In particular, the residuals from previous layer, discrete relation (ReLU activation), quadratic relation, and non-linear relation (Tanh and sigmoid) were used for linear and non-linear feature transformation.[5] The non-linear feature interaction layer was defined as:

$$\begin{aligned} X_{l+1} = {} & \mathrm{dropout}(X_l) + \mathrm{dropout}(\mathrm{relu}(W_r X_l)) + \mathrm{dropout}((W_{m1} X_l) \circ (W_{m2} X_l)) \\ & + \mathrm{dropout}(\tanh(W_t X_l) \circ \sigma(W_s X_l)) \end{aligned} \quad (7)$$

where X_l represented features after linear and non-linear feature transformation in the *l-1* (l = 1, 2, 3, 4, ……) layer; W_r represented the parameter of ReLU activation; W_{m1}, W_{m2}, represented the parameter of quadratic relation; W_t and W_s represented the parameter of Tanh and sigmoid respectively. The DL framework was constructed using Keras 2.3.1 + tensorflow1.14.0 (GPU acceleration with one Nvidia RTX 2080Ti graphical card). Classification was performed using a 10-fold cross-validation (repeated for 20 rounds) to assess the diagnostic accuracy within the discovery cohorts. For the permutation test, we randomly permuted disease label ("0" for healthy controls and "1" for patients, previously) for 1000 times and calculated the distribution of AUC using the uninformative data obtained by random permutation.

Machine learning (ML) method: All ML in this work were performed based on Python 3.6 (Anaconda distribution). The least absolute shrinkage and selector operator (LASSO), random forest (RF)[6] and support vector machine (SVM)[6b, 7] were implemented in Python Scikit-learn 0.22.2. For orthogonal partial least squares discriminant analysis (OPLS-DA),[8] the algorithm was derived from PLS-DA algorithms, for the purpose of improving model interpretation and reducing model complexity. For valid comparison, we performed 20 rounds of 10-fold cross-validation for all ML methods, strictly following the same experimental configuration with the preconstructed DL method.

Computer-assisted diagnosis: The performance of classification models constructed by both DL and ML was measured by sensitivity, specificity, and area under curve (AUC). Receiver operating characteristic (ROC) curves were generated using classification probabilities of stroke patients versus healthy controls and the true labels of each test mass spectrum. The final AUC was an average of all the AUCs obtained from all folds of the cross-validation. Sensitivity and specificity were determined by dividing the total number of correctly labeled stroke patients and the total number of correctly labeled healthy controls, respectively, by the total number of all samples. Notably, the validation cohort was independent of the discovery cohort in the diagnosis stage for blind testing.

Biomarker selection: The significant variables by DL were identified and selected by activation maximization (AM). Typically, each SMF (mass spectrum) was converted to a two-dimensional graphic.[9] All graphics were listed in sequence, shown as a saliency map (Figure

S5) with discriminant significance for each input feature towards feature selection. The conversion was mainly dependent on Keras Visualization Toolkit (https://github.com/raghakot/keras-vis/blob/master/README.md). Top 20 m/z signals were selected with score over 0.2 (the discriminant saliency derived from saliency map), contributing the most to the differentiation between healthy controls and stroke patients. The as-selected 20 m/z signals were validated as metabolite feature panel by accurate mass measurement, according to the human metabolome database (HMDB, http://www.hmdb.ca/). The differential metabolomic profiles reflecting their respective biochemical pathways were analysed by pathway topology analysis using MetaboAnalyst (http://www.metaboanalyst.ca/).

Statistical analysis: Peak extraction, alignment, normalization, and standardization were performed using a "home-built" code by MATLAB (R2016a, The Mathworks, Natick, MA). Other univariate statistical analyses in this work were performed using SPSS software version 19.0 (IBM Corp., Armonk, New York), including one-sided DeLong test for AUC comparison, two-sided Student's t-test for age comparison, and Chi-square test for sex comparison. All significance level was set as 5%. The similarity score between two mass spectra was calculated by cosine correlation method in python following a reported algorithm.[10] To ensure to reach a statistical power > 0.9, power analysis was performed based on the pilot study by PASS (NCSS, version 2020) before experiments. Estimation was based on a reference AUC as 0.5 with the level of significance set at 0.05 and a statistical power of 0.9. Figures were prepared using GraphPad Prism (GraphPad) and Origin software (OriginLab). Data were shown as the mean ± s.d. with n = 172/172 (controls/patients) for clinical indexes.

Reference

[1] W. J. Powers, A. A. Rabinstein, T. Ackerson, O. M. Adeoye, N. C. Bambakidis, K. Becker, J. Biller, M. Brown, B. M. Demaerschalk, B. Hoh, *Stroke* **2018**, *49*, e46.

[2] a) R. Zhang, B. Le, W. Xu, K. Guo, X. Sun, H. Su, L. Huang, J. Huang, T. Shen, T. Liao, Y. Liang, J. X. J. Zhang, H. Dai, K. Qian, *Small Methods* **2019**, *3*, 1800474; b) B. Liu, Y. Li, H. Wan, L. Wang, W. Xu, S. Zhu, Y. Liang, B. Zhang, J. Lou, H. Dai, K. Qian, *Adv. Funct. Mater.* **2016**, *26*, 7994.

[3] a) X. Sun, L. Huang, R. Zhang, W. Xu, J. Huang, D. D. Gurav, V. Vedarethinam, R. Chen, J. Lou, Q. Wang, J. Wan, K. Qian, *ACS Central Sci.* **2018**, *4*, 223; b) L. Huang, J. Wan, X. Wei, Y. Liu, J. Huang, X. Sun, R. Zhang, D. D. Gurav, V. Vedarethinam, Y. Li, R. Chen, K. Qian, *Nat. Commun.* **2017**, *8*, 220; c) J. Yang, R. Wang, L. Huang, M. Zhang, J. Niu, C. Bao, N. Shen, M. Dai, Q. Guo, Q. Wang, Q. Wang, Q. Fu, K. Qian, *Angew. Chem. Int. Edit.* **2019**, *59*, 1703.

[4] T. Pan, J. Chen, Z. Zhou, C. Wang, S. He, *IEEE T. Ind. Inform.* **2019**, *15*, 5119.

[5] N. Srivastava, G. Hinton, A. Krizhevsky, I. Sutskever, R. Salakhutdinov, *J. Mach. Learn. Res.* **2014**, *15*, 1929.

[6] a) M. Reichstein, G. Camps-Valls, B. Stevens, M. Jung, J. Denzler, N. Carvalhais, Prabhat, *Nature* **2019**, *566*, 195; b) F. Pedregosa, G. Varoquaux, A. Gramfort, V. Michel, B. Thirion, O. Grisel, M. Blondel, P. Prettenhofer, R. Weiss, V. Dubourg, J. Vanderplas, A. Passos, D. Cournapeau, M. Brucher, M. Perrot, E. Duchesnay, *J. Mach. Learn. Res.* **2011**, *12*, 2825.

[7] A. Soualhi, K. Medjaher, N. Zerhouni, *IEEE Trans. Instrum. Meas.* **2015**, *64*, 52.

[8] a) J. Boccard, D. N. Rutledge, *Anal. Chim. Acta* **2013**, *769*, 30; b) J. A. Westerhuis, E. J. J. van Velzen, H. C. J. Hoefsloot, A. K. Smilde, *Metabolomics* **2010**, *6*, 119.

[9] Z. Li, *Trends Cogn. Sci.* **2002**, *6*, 9.

[10] a) K. X. Wan, I. Vidavsky, M. L. Gross, *J. Am. Soc. Mass Spectr.* **2002**, *13*, 85; b) Y. Zhu, L. Qiao, M. Prudent, A. Bondarenko, N. Gasilova, S. B. Moller, N. Lion, H. Pick, T. Gong, Z. Chen, P. Yang, L. T. Loveyf, H. H. Girault, *Chem. Sci.* **2016**, *7*, 2987.

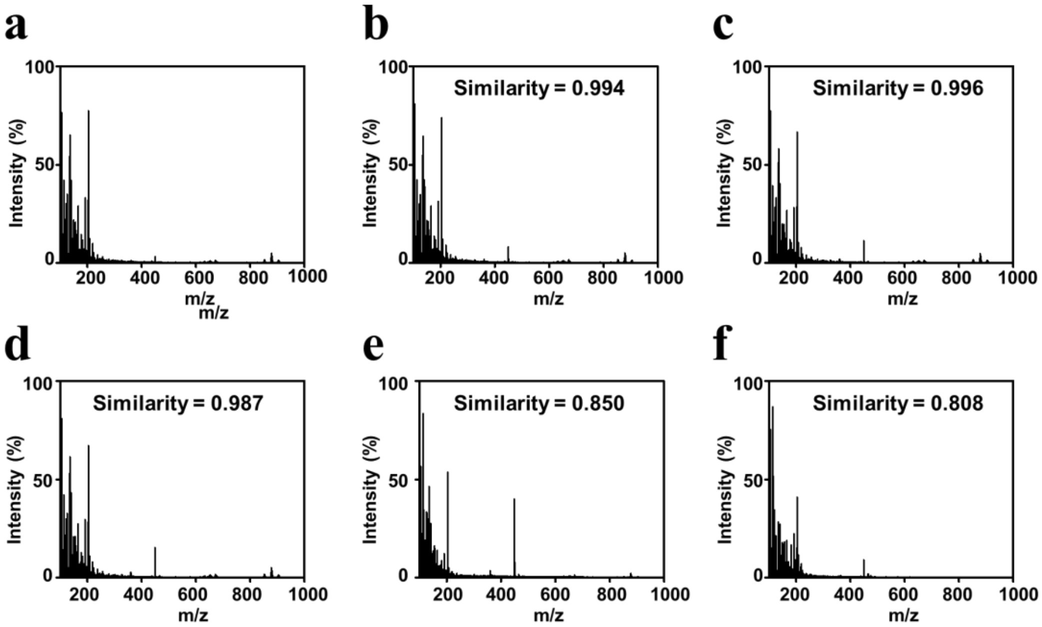

Figure S1. Typical mass spectra from a healthy control by nano-assisted LDI mass spectrometry (MS), consuming (a) 100 nL, (b) 50 nL, (c) 150 nL, (d) 200 nL, (e) 500 nL, and (f) 1000 nL of native serum.

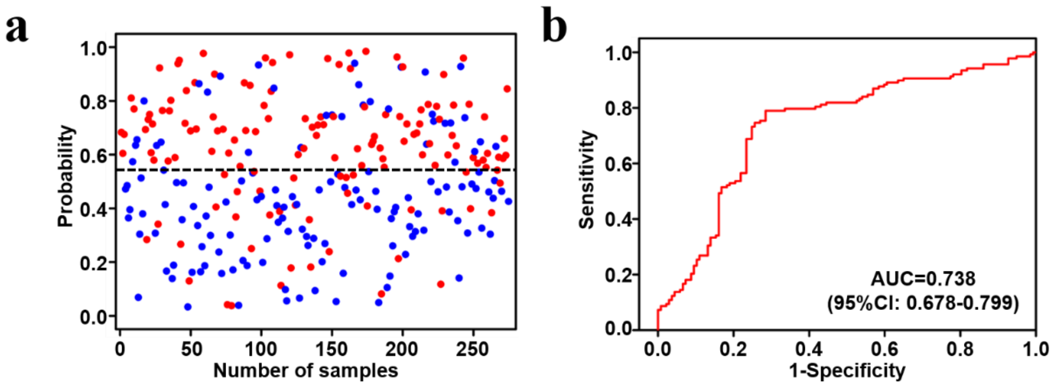

Figure S2. Serum metabolic fingerprint (SMF) based diagnosis using deep learning (DL) in the discovery cohort, by stroke network (SN) model. (a) A sample-level plot stratifying healthy controls (blue) and stroke patients (red) for discovery cohort (*n* = 275; 137/138, controls/patients). The black dashed line indicated the threshold, determined by maximized the Youden index in the discovery cohort. (b) ROC curve showing the diagnostic performance of SN for the discovery cohort, with average AUC of 0.738 (95% CI: 0.678-0.799).

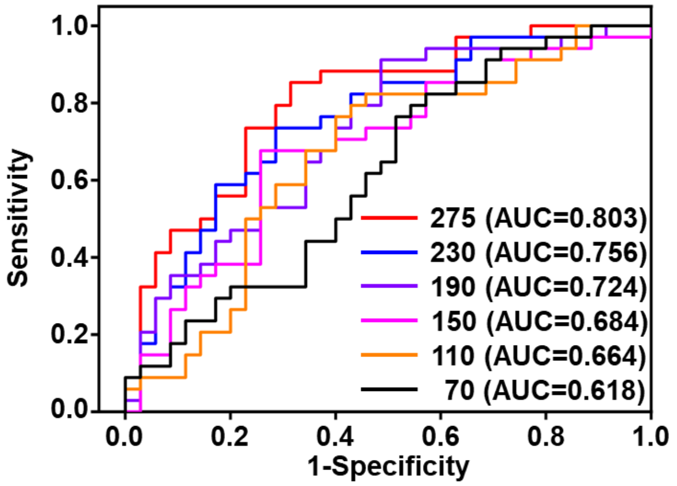

Figure S3. Averaged ROC curves with AUC to optimize the number of discovery cohort, analysing from 70 (35/35, control/patients) to 275 (137/138, control/patients).

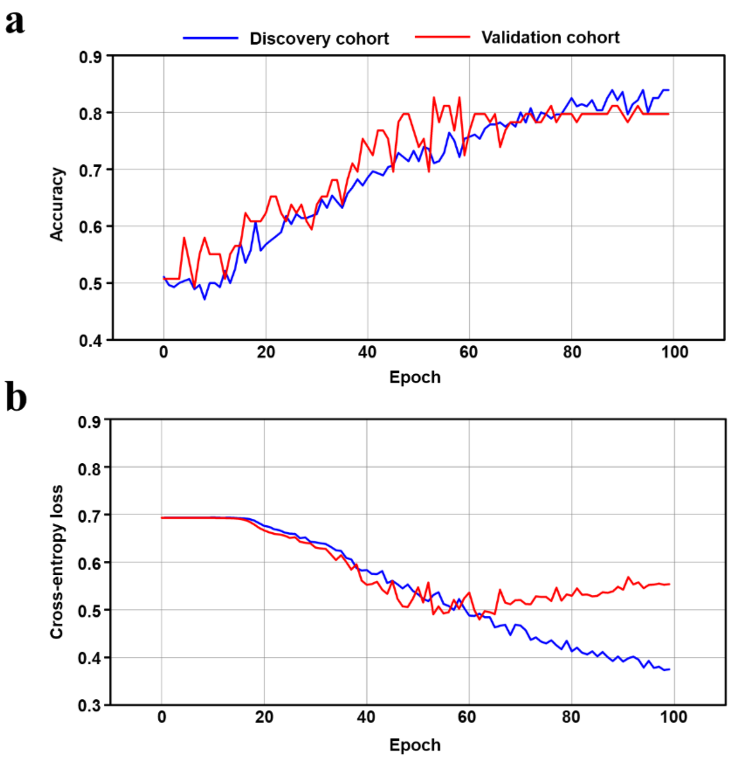

Figure S4. Epoch optimization of SN. (a) Tensorboard accuracy and (b) cross-entropy loss from the discovery cohort (blue) and validation cohort (red), to optimize the number of training steps (epoch) from 0 to 100.

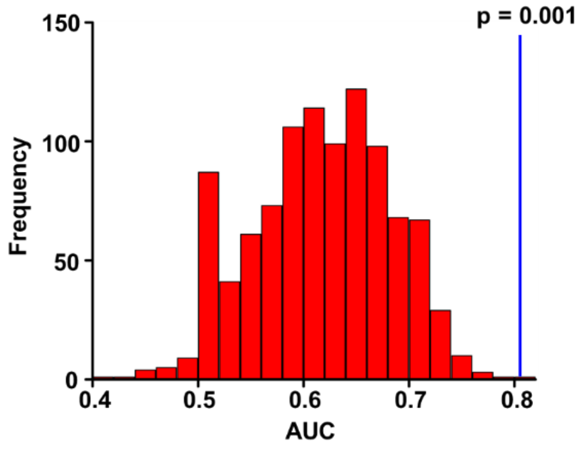

Figure S5. Distribution of AUC calculated using the uninformative data obtained by random permutation (1000 permutations).

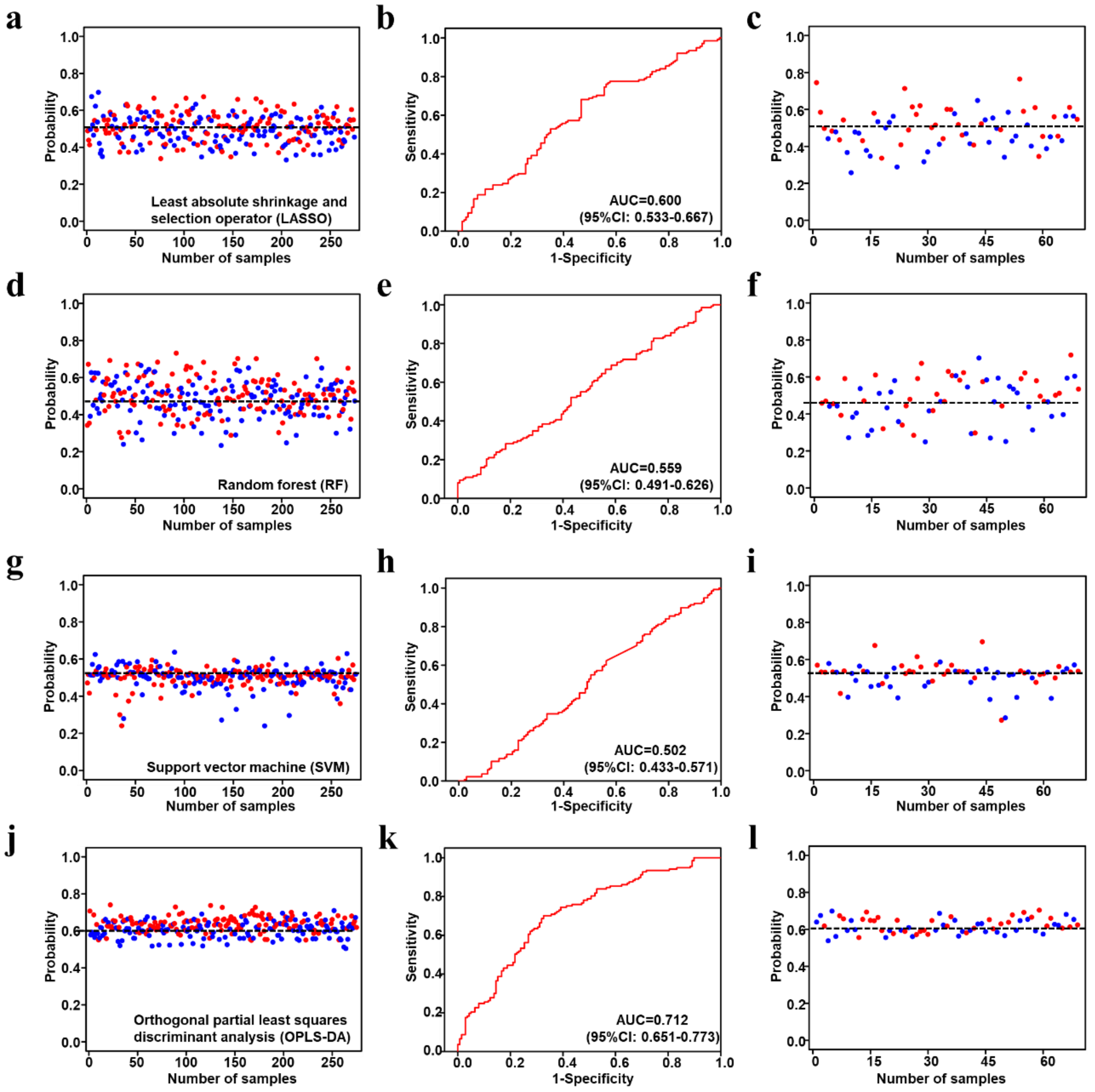

Figure S6. Diagnosis of stroke by major clinical used machine learning (ML) algorithms, including (a-c) least absolute shrinkage and selector operator (LASSO), (d-f) random forest (RF), (g-i) support vector machine (SVM), and (j-l) orthogonal partial least squares discriminant analysis (OPLS-DA). A sample-level plot by (a) LASSO, (d) RF, (g) SVM, and (j) OPLS-DA, stratifying healthy controls (blue) and stroke patients (red) for discovery cohort (*n* = 275; 137/138, controls/stroke patients). ROC curves showing the diagnostic performance of ML for discovery cohort, with average AUC of 0.600 (95% CI: 0.533-0.667) for (b) LASSO, 0.559 (95% CI: 0.491-0.626) for (e) RF, 0.502 (95% CI: 0.433-0.571) for (h) SVM, and 0.712 (95% CI: 0.651-0.773) for (k) OPLS-DA. The black dashed lines in sample-level plot indicated the threshold, determined by maximized the Youden index in the discovery cohort. A sample-level plot by (c) LASSO, (f) RF, (i) SVM, and (l) OPLS-DA, stratifying healthy controls (blue) and stroke patients (red) for validation cohort (*n* = 69; 35/34, controls/stroke patients). The black dashed lines in sample-level plot indicated the threshold, determined by maximized the Youden index in the validation cohort.

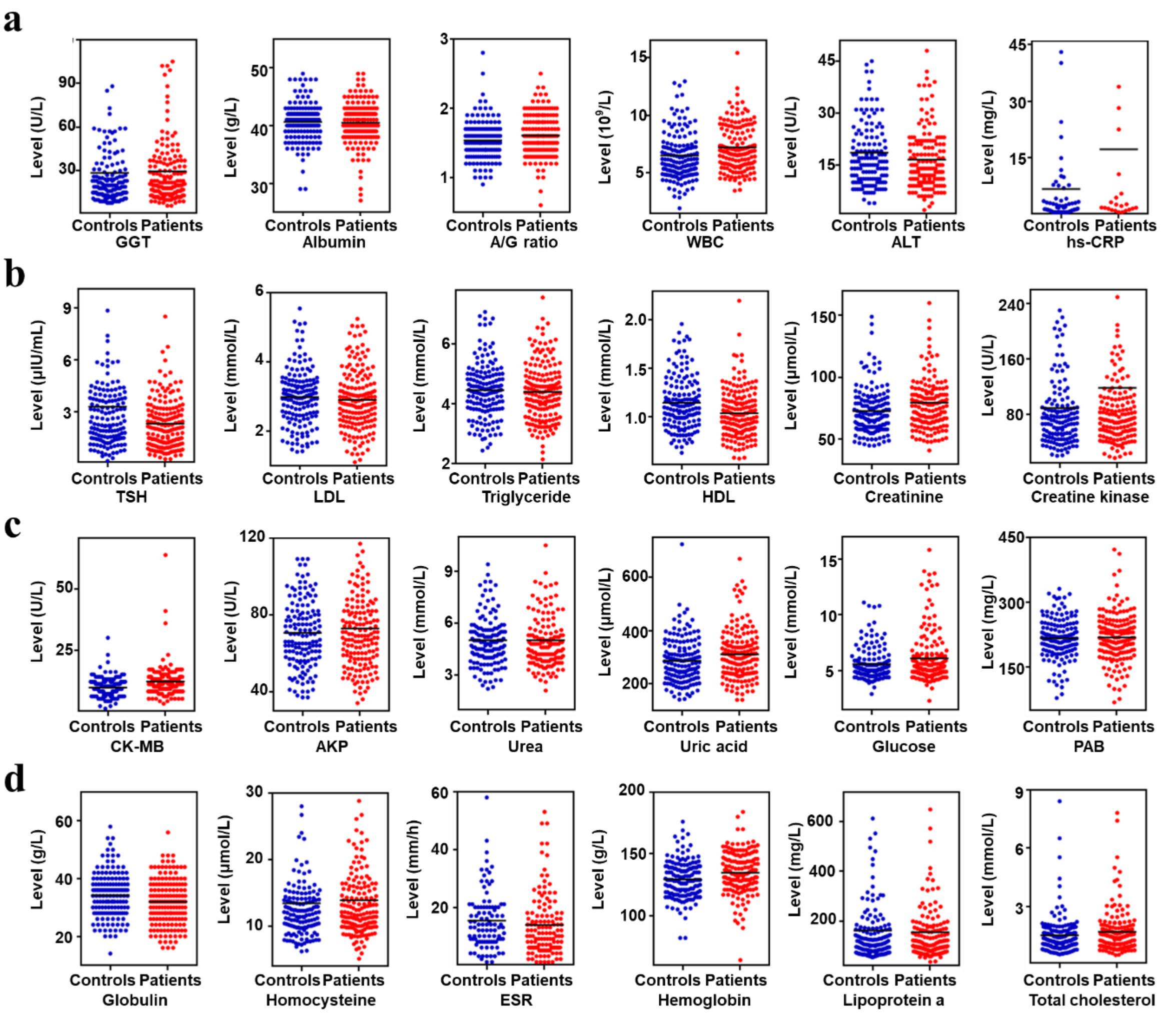

Figure S7. Scatter plot for blood level of 24 clinical indexes for 172 healthy controls (blue) and 172 stroke patients (red), including (a) γ-glutamyl transferase (GGT), Albumin, Albumin/globulin (A/G) ratio, White blood cell count (WBC), Alanine aminotransferase (ALT), Hypersensitive C-reactive protein (hs-CRP); (b) Thyroid Stimulating Hormone (TSH), Low density lipoprotein (LDL), Triglyceride, High density lipoprotein (HDL), Creatinine, Creatine kinase; (c) Creatine kinase isoenzymes (CK-MB), Alkaline phosphatase (AKP), Urea, Uric acid, Glucose, Prealbumin (PAB); and (d) Globulin, Homocysteine, Erythrocyte sedimentation rate (ESR), Hemoglobin, Lipoprotein a, Total cholesterol.

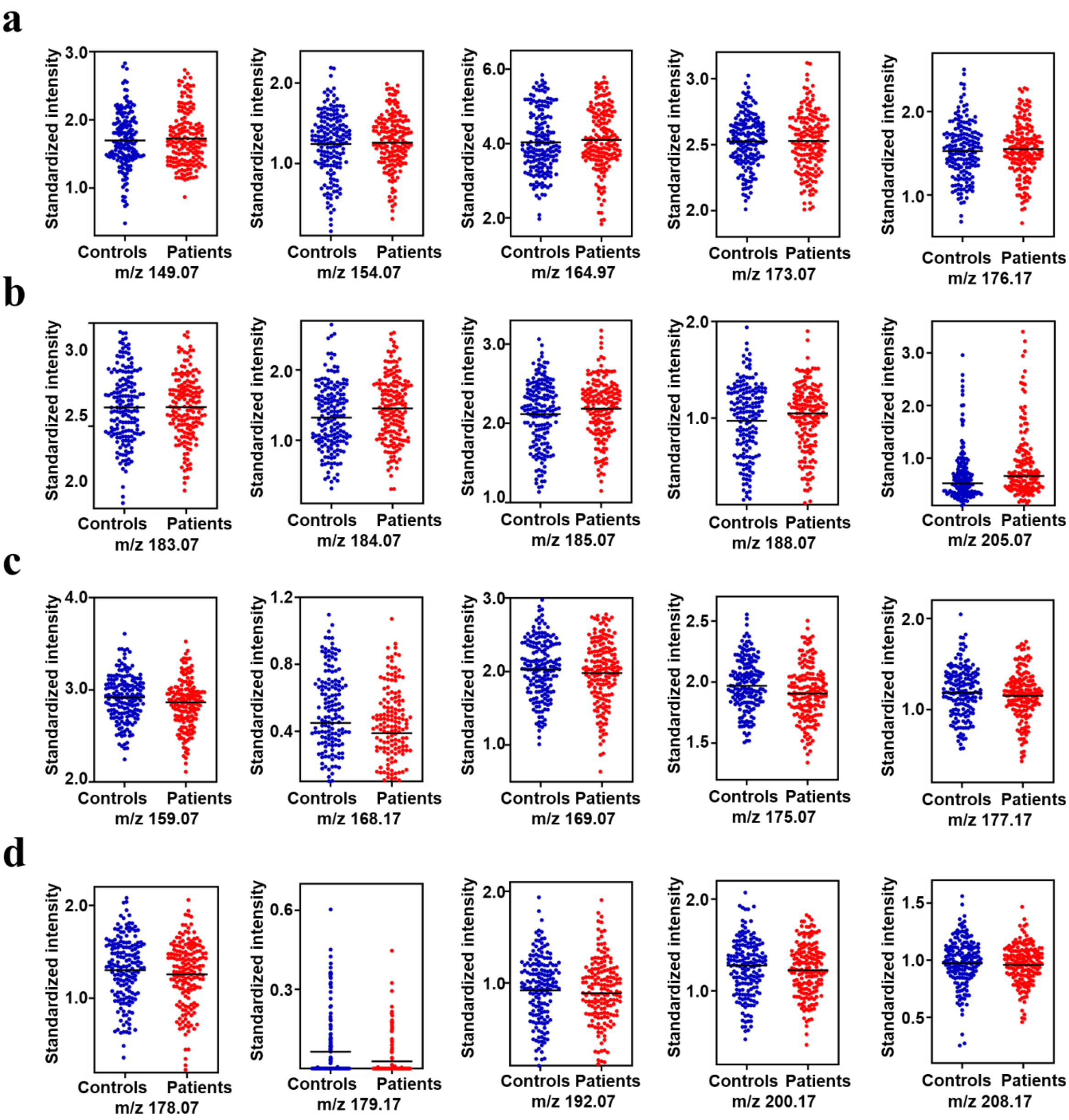

Figure S8. Differential regulation of 20 key metabolite features. Scatter plots for z score standardized signal intensities of 20 metabolite features for 172 healthy controls (blue) and 172 stroke patients (red).

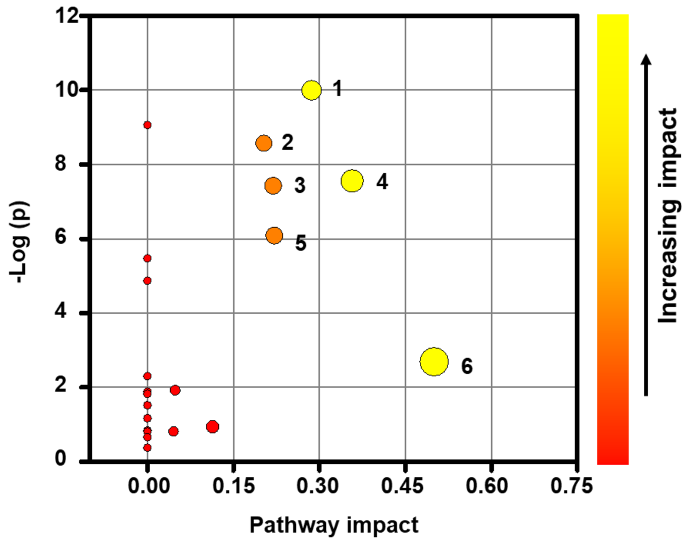

Figure S9. Potential pathways differentially regulated in stroke patients, compared to healthy controls. The 20 selected metabolite features were tested to identify altered pathways. The colour and size of each circle were correlated to the *p* value and pathway impact value. A total of six pathways were differentially regulated: (1) lysine degradation, (2) tyrosine metabolism, (3) histidine metabolism, (4) phenylalanine metabolism, (5) pentose and glucuronate interconversions, and (6) phenylalanine, tyrosine, and tryptophan biosynthesis. Pathways with impact value > 0.15 and $p < 0.05$ were considered to be differentially altered between stroke patients and healthy controls.

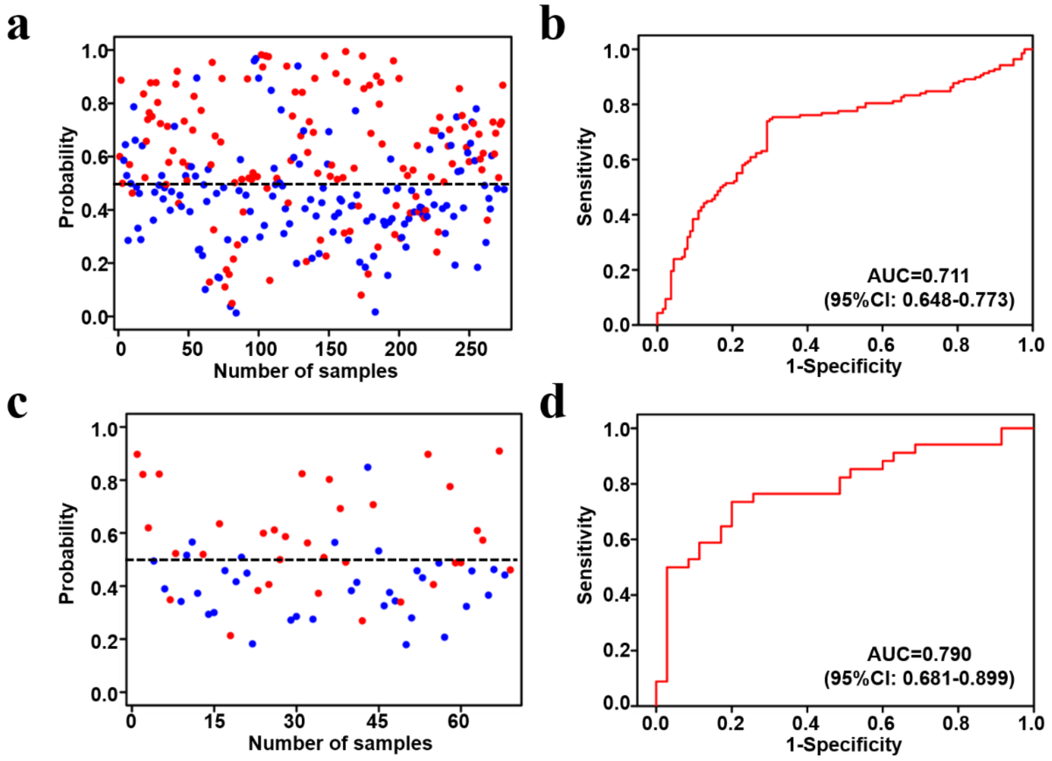

Figure S10. 20 key metabolite features based diagnosis using deep learning (DL) in the (a&b) discovery and (c&d) validation cohorts, by a SN model. A sample-level plot stratifying controls (blue) and stroke patients (red) for (a) discovery cohort (*n* = 275; 137/138, controls/patients); and (c) validation cohort (*n* = 69; 35/34, controls/stroke patients). The black dashed lines in sample-level plot indicated the threshold, determined by maximized the Youden index in the discovery cohort. ROC curve showing the diagnostic performance of SN for (b) discovery cohort with average AUC of 0.711 (95% CI: 0.648-0.773); and (d) validation cohort with average AUC of 0.790 (95% CI: 0.681-0.899).

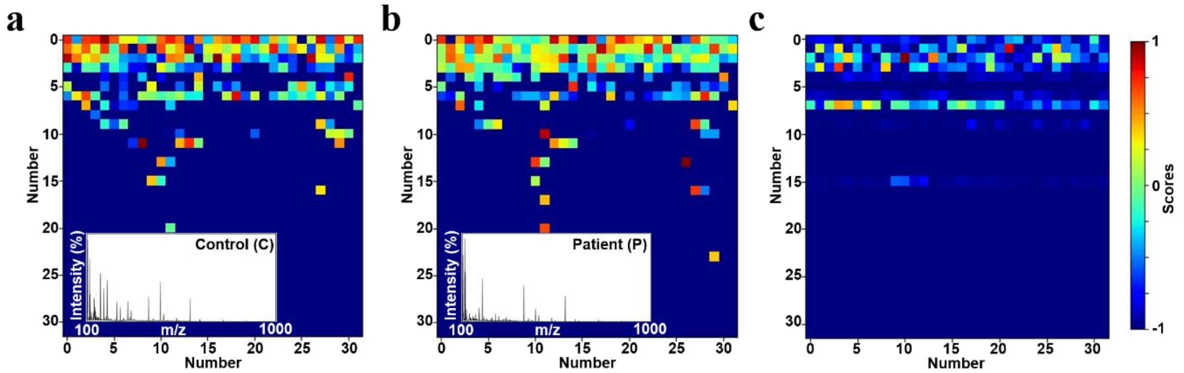

Figure S11. Feature selection by DL. Two dimensional graphics of (a) a healthy control and (b) a stroke patient, converted from corresponding SMFs (inset). (c) Saliency map consisted of the discriminant significance for each input feature (1024 features in total). The color scale ranging from -1 (blue) to 1 (red) indicated normalized mass spectrometric signal intensity in (a&b) and significant weight in (c), from low to high.

Table S1: Clinical characteristics of controls and stroke patients for discovery and validation cohorts.

Characteristics		Discovery			Validation		
		Controls (*n* = 137)	Patients (*n* =138)	*P* value	Controls (*n* =35)	Patients (n=34)	P value
Age (mean (range))		67.90 (39-90)	68.52 (32-94)	0.696[a]	67.77 (33-90)	67.03 (33-93)	0.829[a]
Sex							
	Male	58	53	0.572[b]	14	15	0.888[b]
	Female	80	84		20	20	

[a)] *P* value was calculated by two-sided Student's t-test; [b)] *P* value was calculated by Chi-square test.

Table S2: Baseline characteristics of clinical indexes.

Index	Cohort	N	Mean	SD
γ -glutamyl transferase (GGT)	Controls	172	28.49	30.325
	Patients	172	29.75	25.600
Albumin	Controls	172	40.64	3.398
	Patients	172	40.51	3.652
Albumin/globulin (A/G) ratio	Controls	172	1.53	0.280
	Patients	172	1.60	0.303
White blood cell count (WBC)	Controls	172	6.51	2.252
	Patients	172	7.25	2.176
Alanine aminotransferase (ALT)	Controls	172	18.54	10.998
	Patients	172	16.67	9.718
Hypersensitive C-reactive protein (hs-CRP)	Controls	172	8.13	16.028
	Patients	172	19.13	46.646
Thyroid Stimulating Hormone (TSH)	Controls	172	3.28	5.009
	Patients	172	2.27	1.399
Low density lipoprotein (LDL)	Controls	172	2.98	0.828
	Patients	172	2.87	0.863
Triglyceride	Controls	172	1.15	1.011
	Patients	172	1.68	1.086
High density lipoprotein (HDL)	Controls	172	1.14	0.279
	Patients	172	1.03	0.253
Creatinine	Controls	172	72.82	19.009
	Patients	172	78.64	23.689
Creatine kinase	Controls	172	89.21	70.272
	Patients	172	118.96	197.147
Creatine kinase isoenzymes (CK-MB)	Controls	172	9.63	3.854
	Patients	172	12.09	6.236
Alkaline phosphatase (AKP)	Controls	172	70.56	18.632
	Patients	172	73.00	21.814
Urea	Controls	172	5.01	1.600
	Patients	172	4.98	1.801
Uric acid	Controls	172	285.63	85.677
	Patients	172	307.20	92.102
Glucose	Controls	172	5.58	1.453
	Patients	172	6.06	2.235
Prealbumin (PAB)	Controls	172	216.25	47.732

	Patients	172	219.10	56.297
Globulin	Controls	172	27.07	3.955
	Patients	172	26.00	4.457
Homocysteine	Controls	172	13.42	7.184
	Patients	172	13.84	7.485
Erythrocyte sedimentation rate (ESR)	Controls	172	15.33	9.997
	Patients	172	13.79	10.763
Hemoglobin	Controls	172	129.29	15.320
	Patients	172	134.58	17.228
Lipoprotein a	Controls	172	160.90	174.445
	Patients	172	151.26	137.476
Total cholesterol	Controls	172	4.45	0.945
	Patients	172	4.37	0.955

Table S3: Clinical indexes for differentiating stroke patients from healthy controls.

Clinical index	*P* value[a]	AUC (95% CI)[b]
γ-glutamyl transferase (GGT)	0.7396	0.518 (0.332-0.704)
Albumin	0.7259	0.328 (0.161-0.495)
Albumin/globulin ratio	0.0342	0.541 (0.345-0.737)
White blood cell count (WBC)	0.0051	0.454 (0.261-0.647)
Alanine aminotransferase (ALT)	0.0947	0.462 (0.271-0.652)
Hypersensitive C-reactive protein (hs-CRP)	0.1034	0.565 (0.381-0.749)
Thyroid Stimulating Hormone (TSH)	0.0171	0.447 (0.245-0.649)
Low density lipoprotein (LDL)	0.3948	0.475 (0.286-0.663)
Triglyceride	0.1419	0.570 (0.356-0.784)
High density lipoprotein (HDL)	0.0003	0.337 (0.172-0.501)
Creatinine	0.0096	0.532 (0.353-0.712)
Creatine kinase	0.0907	0.561 (0.358-0.765)
Creatine kinase isoenzymes (CK-MB)	<0.0001	0.524 (0.325-0.723)
Alkaline phosphatase (AKP)	0.3271	0.523 (0.327-0.719)
Urea	0.9841	0.440 (0.260-0.619)
Uric acid	0.0175	0.632 (0.469-0.795)
Glucose	0.0190	0.346 (0.177-0.516)
Prealbumin (PAB)	0.6760	0.394 (0.205-0.584)
Globulin	0.0221	0.350 (0.159-0.541)
Homocysteine	0.5780	0.353 (0.155-0.552)
Erythrocyte sedimentation rate (ESR)	0.2733	0.549 (0.360-0.738)
Hemoglobin	0.0042	0.484 (0.292-0.676)
Lipoprotein a	0.7144	0.531 (0.341-0.721)
Total cholesterol	0.6179	0.452 (0.274-0.630)

[a)] *P* value was calculated by two-sided Student's t-test; [b)] AUC was acquired by univariate ROC curve analysis for individual biomarker.

Table S4: m/z signals selected as key metabolite features for differentiating stroke patients from healthy controls.

ID	m/z	Score[a]	*P* value[b]	AUC (95% CI)[c]	Potential biomarkers	Regulated expression
1	149.07	0.2613	0.5630	0.502 (0.440-0.563)	Imidazoleacetic acid	↑
2	154.07	0.2092	0.7395	0.505 (0.444-0.566)	L-Isoleucine/L-Leucine	↑
3	159.07	0.2090	0.0161	0.427 (0.366-0.487)	Phenylacetic acid	↓
4	164.97	0.2210	0.4518	0.535 (0.474-0.596)	Sumiki's acid	↑
5	168.17	0.3127	0.0041	0.416 (0.355-0.476)	4-Trimethylammoniobutanoic acid	↓
6	169.07	0.4986	0.3127	0.476 (0.415-0.537)	L-Lysine/L-Glutamine/D-Glutamine/3-Oxoglutaric acid	↓
7	173.07	0.4430	0.9334	0.496 (0.435-0.557)	L-Arabinose/L-Xylulose/D-Xylulose/D-Xylose	↑
8	175.07	0.8393	0.0040	0.411 (0.351-0.471)	ortho-Hydroxyphenylacetic acid/p-Hydroxyphenylacetic acid	↓
9	176.17	0.2402	0.5135	0.519 (0.458-0.580)	Dopamine	↑
10	177.17	0.2447	0.2482	0.457 (0.396-0.518)	Gentisic acid	↓
11	178.07	0.4277	0.2534	0.462 (0.401-0.523)	L-Histidine	↓
12	179.17	0.2666	0.0007	0.432 (0.371-0.0.492)	Orotic acid	↓
13	183.07	0.2262	0.8001	0.507 (0.446-0.569)	Oxoadipic acid	↑
14	184.07	0.3237	0.0173	0.574 (0.514-0.634)	Aminoadipic acid	↑
15	185.07	0.2228	0.1081	0.540 (0.479-0.601)	5-Hydroxylysine	↑
16	188.07	0.2955	0.5879	0.507 (0.446-0.568)	L-Phenylalanine	↑
17	192.07	0.2805	0.4589	0.466 (0.404-0.527)	3-Methylhistidine	↓
18	200.17	0.4452	0.6145	0.483 (0.422-0.545)	5-Hydroxytryptophol	↓
19	205.07	0.2913	0.0002	0.605 (0.546-0.665)	Homovanillic acid	↑
20	208.17	0.2453	0.4581	0.468 (0.407-0.529)	DL-O-Phosphoserine	↓

[a)] Score referring to dicriminant saliency from saliency map; [b)] *P* value was calculated by two-sided Student's t-test; [c)] AUC was acquired by univariate ROC curve analysis for the individual biomarker.

Table S5: Pathways regulated in stroke patients and healthy controls.

Pathway Hit[a]	*P* value[b]	-Log (p)	Impact[c]
Lysine degradation	4.56E-05	9.9959	0.28
Aminoacyl-tRNA biosynthesis	0.0001	9.0619	0
Pentose and glucuronate interconversions	0.0001	8.5708	0.20
Phenylalanine metabolism	0.0005	7.5577	0.35
Tyrosine metabolism	0.0006	7.4267	0.21
Histidine metabolism	0.0023	6.0872	0.22
D-Glutamine and D-glutamate metabolism	0.0042	5.4726	0
Valine, leucine and isoleucine biosynthesis	0.0077	4.8700	0
Phenylalanine, tyrosine and tryptophan biosynthesis	0.0679	2.6891	0.50
Nitrogen metabolism	0.1002	2.3004	0
Pyrimidine metabolism	0.1461	1.9231	0.05
Valine, leucine and isoleucine degradation	0.1522	1.8823	0
Biotin metabolism	0.1615	1.8227	0
Arginine biosynthesis	0.2189	1.5191	0
beta-Alanine metabolism	0.3102	1.1703	0
Alanine, aspartate and glutamate metabolism	0.3912	0.9383	0.11
Glyoxylate and dicarboxylate metabolism	0.4333	0.8361	0
Cysteine and methionine metabolism	0.4434	0.8131	0
Glycine, serine and threonine metabolism	0.4434	0.8131	0.04
Tryptophan metabolism	0.5181	0.6575	0
Purine metabolism	0.6886	0.3730	0

a) The pathway analysis was performed on MetaboAnalyst using the website built-in function; b) *P* value was calculated from the pathway topology analysis; c) Impact was calculated from pathway topology analysis.
